# Manual therapies for migraine: a systematic review

**DOI:** 10.1007/s10194-011-0296-6

**Published:** 2011-02-05

**Authors:** Aleksander Chaibi, Peter J. Tuchin, Michael Bjørn Russell

**Affiliations:** 1 Head and Neck Research Group, Research Centre, Akershus University Hospital, 1478 Lørenskog, Norway; 2 Department of Chiropractic, Macquarie University, Sydney, NSW 2109 Australia; 3 Institute of Clinical Medicine, Akershus University Hospital, University of Oslo, 1474 Nordbyhagen, Norway

**Keywords:** Manual therapies, Massage, Physiotherapy, Chiropractic, Migraine, Treatment

## Abstract

Migraine occurs in about 15% of the general population. Migraine is usually managed by medication, but some patients do not tolerate migraine medication due to side effects or prefer to avoid medication for other reasons. Non-pharmacological management is an alternative treatment option. We systematically reviewed randomized clinical trials (RCTs) on manual therapies for migraine. The RCTs suggest that massage therapy, physiotherapy, relaxation and chiropractic spinal manipulative therapy might be equally effective as propranolol and topiramate in the prophylactic management of migraine. However, the evaluated RCTs had many methodological shortcomings. Therefore, any firm conclusion will require future, well-conducted RCTs on manual therapies for migraine.

## Introduction

Migraine is usually managed by medication, but some patients do not tolerate acute and/or prophylactic medicine due to side effects, or contraindications due to co-morbidity of myocardial disorders or asthma among others. Some patients wish to avoid medication for other reasons. Thus, non-pharmacological management such as massage, physiotherapy and chiropractic may be an alternative treatment option. Massage therapy in Western cultures uses classic massage, trigger points, myofascial release and other passive muscle stretching among other treatment techniques which are applied to abnormal muscle tissue. Modern physiotherapy focuses on rehabilitation and exercise, while manual treatment emphasis postural corrections, soft tissue work, stretching, active and passive mobilization and manipulation techniques. Mobilization is commonly defined as movement of joints within the physiological range of motion [1]. The two most common chiropractic techniques are the diversified and Gonstead, which are used by 91 and 59% of chiropractors [2]. Chiropractic spinal manipulation (SM) is a passive-controlled maneuver which uses a directional high-velocity, low-amplitude thrusts directed at a specific joint past the physiological range of motion, without exceeding the anatomical limit [1]. The application and duration of the different manual treatments varies among those who perform it. Thus, manual treatment is not necessarily as uniform as, for instance, specific treatment with a drug in a certain dose.

This paper systematically review randomized controlled trials (RCTs) assessing the efficacy of manual therapies on migraine, i.e., massage, physiotherapy and chiropractic.

## Method

The literature search was done on CINAHL, Cochrane, Medline, Ovid and PubMed. Search words were migraine and chiropractic, manipulative therapy, massage therapy, osteopathic treatment, physiotherapy or spinal mobilization. All RCTs written in English using manual therapy on migraine were evaluated. Migraine was preferentially classified according to the criteria of the International Headache Societies from 1988 or its revision from 2004, although it was not an absolute requirement [3, 4]. The studies had to evaluate at least one migraine outcome measure such as pain intensity, frequency, or duration. The methodological quality of the included RCT studies was assessed independently by the authors. The evaluation covered study population, intervention, measurement of effect, data presentation and analysis (Table 1). The maximum score is 100 points and ≥50 points considered to be methodology of good quality [5–7].

**Table 1:** Criteria list of methodological quality assessment of randomized controlled trials (RCTs) [7]

1.	*Study population* (30 points)
(a)	Description of inclusion and exclusion criteria (1 point). Restriction to a homogeneous study population (1 point)
(b)	Comparability of relevant baseline characteristics: duration of complaint (1 point), value of outcome measures (1 point), age (1 point), recurrences (1 point), and radiating complaints/associated symptoms (1 point)
(c)	Description of the randomization procedure (2 points). Randomization procedure which excluded bias, i.e., random numbers table (2 points)
(d)	Description of dropouts for each group and their reasons (3 points)
(e)	Loss to follow-up: <20% loss to follow-up (2 points), or <10% loss to follow-up (4 points)
(f)	Sample size: >50 subjects in the smallest group after randomization (6 points), or >100 subjects in the smallest group after randomization (12 points)
2.	*Interventions* (30 points)
(g)	Correct description of the manual intervention (5 points). All interventions described (5 points)
(h)	Pragmatic study: comparison with an existing treatment modality (5 points)
(i)	Co-interventions avoided in the design of the study (5 points)
(j)	Comparison with a placebo control group (5 points)
(k)	Mention of the experience of the therapist (5 points)
3.	*Measurement of effect* (30 points)
(l)	Placebo controlled studies: patients blinded (3 points), blinding evaluated and fully successful (2 points) or pragmatic studies: patients fully naive, evaluated and fully successful (3 points), time restriction of no manual treatments for at least 1 year (2 points)
(m)	Outcome measures: pain assessment (2 points), global measure of improvement (2 points), functional status (2 points), spinal mobility (2 points), medical consumption (2 points)
(n)	Each blinded outcome measure mentioned under item M earns 2 points
(o)	Analysis of post-treatment data (3 points), inclusion of a follow-up period longer than 6 months (2 points)
4.	*Data presentation and analysis* (10 points)
(p)	Intention-to-treat analysis when loss to follow-up is <10% or intention-to-treat analysis as well as worst-case analysis for missing values when loss to follow-up is >10% (5 points)
(q)	Corrected presentation of the data: mean or median with a standard deviation or percentiles for continuous variables (5 points)

## Results

The literature search identified seven RCT on migraine that met our inclusion criteria, i.e., two massage therapy studies [8, 9], one physiotherapy study [10] and four chiropractic spinal manipulative therapy studies (CSMT) [11–14], while we found no RCTs studies on spinal mobilization or osteopathic as a intervention for migraine.

### Methodological quality of the RCTs

Table 2 shows the authors average methodological score of the included RCT studies [8–14]. The average score varied from 39 to 59 points. Four RCTs were considered to have a good quality methodology score (≥50), and three RCTs had a low score.

**Table 2:** Quality score of the analyzed randomized controlled trials (RCTs) using manual therapies for treatment of migraine

Study	a	b	c	d	e	f	g	h	i	j	k	l	m	n	o	p	q	Total
Hernandez [8]	2	2	4	0	0	0	10	0	5	0	5	0	6	0	0	0	5	39
Lawler [9]	2	3	4	3	4	0	10	0	5	0	5	0	6	0	3	5	5	55
Marcus [10]	2	3	2	3	4	0	10	5	0	0	0	0	6	0	5	5	5	50
Parker [11, 12]	2	5	2	3	4	0	10	5	5	0	0	0	6	0	3	0	0	45
Nelson [13]	2	4	4	3	0	6	10	5	0	0	5	0	6	0	3	0	5	53
Tuchin [14]	2	5	4	3	4	0	10	5	5	0	0	0	8	0	3	5	5	59

The letters corresponds with letters from the criteria list (Table 1)

### Randomized controlled trials

Table 3 shows details and the main results of the different RCT studies [8–14].

**Table 3:** Randomized controlled trials (RCTs) of massage therapy, physical therapy and chiropractic spinal manipulative therapy for migraine

Country	Year	Study population	Participant	Method	Intervention	Results
Massage therapy						
USA [8]	1998	Chronic migraine for at least 6 months diagnosed by a questionnaire Mean years with headache 20.7	26 volunteers Age 24–65 Mean 29.9 years	RCT of 5 weeks duration 5 weeks treatment Questionnaire pre- and post-treatment for intervention and control group Assessment on the first and last day of the 5 weeks study	Massage therapy (*n* = 12) 30 min twice a week focusing on muscle in the neck control group (*n* = 12) not receiving treatment Drop outs (*n* = 2)	Pain intensity was statistically significantly reduced from pre- to post-treatment in the massage group, while the change was not statistically significant in the control group The massage group experienced mean pain intensity was reduced 71% from prior to the first massage and after last massage, while the control groups mean pain intensity was unchanged
New Zealand [9]	2006	Migraineurs diagnosed by questionnaire	48 volunteers (8M, 40F) Age 12–60 years Mean 41.3 years	RCT of 13 weeks duration, i.e., 4 weeks baseline 6 weeks treatment 3 weeks follow-up Comparison of baseline, treatment and follow-up Headache diary recordings	Massage therapy (*n* = 23) 45 min once every week, focusing on neuromuscular and trigger-point framework of the back, shoulders, neck and head Control group (*n* = 23) kept headache diary Drop outs (*n* = 4)	Migraine frequency was significantly reduced in the massage group from baseline to treatment (*p* < 0.01) and baseline to follow-up (*p* < 0.05), while it was unchanged in the control group On average migraine frequency was reduced 34% during treatment and 30% during follow-up in the massage group, while similar figures in the control group was 7 and 2%
Physical therapy						
USA [10]	1998	Migraineurs with at least one migraine attack per week or a total of 5 migraine days per month diagnosed by a neurologist	73 women Age 20–58 years Mean age 37 years	Study 1 RCT of 13.5 months duration, i.e., 2 weeks baseline 4 weeks treatment 3,6,12 months follow-up Comparison of baseline, post-treatment and follow-up	Physical therapy (*n* = 30) Two home sessions daily of about 30 min duration each Relaxation (*n* = 39) Muscle relaxation, breathing exercise and thermal bio feedback. Two home sessions daily of about 20–30 duration each Drop outs (*n* = 4)	The relaxation group had statistically significantly more persons with 50% reduction or more in headache severity than the physical therapy group (*p* < 0.001) 13% (*n* = 4) had 50% reduction or more in mean headache severity in the physical therapy group, i.e., 16% decrease in mean headache severity 51% (*n* = 20) had 50% reduction or more in mean headache severity in the relaxation group, i.e., 41% decrease in mean headache severity
				Follow-up headache recordings at 3, 6 and 12 months on those with 50% reduction or more in mean headache severity	Drop outs at 3, 6 and 12 months follow-up Physical therapy (*n* = 1, 1 and 2) Relaxation (*n* = 2, 4 and 6)	Treatment effect was maintained in both group at 3, 6 and 12 months
		Migraineurs with at least one migraine attack per week or a total of 5 migraine days per month by a neurologist	45 women	Study 2 Participants that did not had a 50% reduction in mean headache severity in study 1 were offered the alternative treatment Comparison of baseline, post-treatment and follow-up	Physical therapy (*n* = 11) Relaxation (*n* = 19) Drop outs (*n* = 15)	55% (6/11) had 50% reduction or more in mean headache severity in physical therapy group, i.e., 30% decrease in mean headache severity 47% (9/19) had 50% reduction or more in mean headache severity in the relaxation group, i.e., 38% decrease in mean headache severity
				Follow-up headache recordings at 3, 6 and 12 months	Drop outs at 3, 6 and 12 months follow-up Physical therapy (*n* = 2, 3 and 3) Relaxation (*n* = 5, 7 and 10)	The relative high number of drop outs makes it difficult to judge the treatment effect at follow-up, but it seems that the effect lasted in the physical therapy group, while it was quite fluctuating in the relaxation group
Chiropractic spinal manipulative therapy (CSMT)						
Australia [11]	1978	Migraineurs diagnosed by a neurologist At least 4 migraine attacks within 2 months	85 volunteers (33M, 52F) Age 12–55 years Mean age 41 years	RCT of 6 months duration, i.e., 2 months baseline 2 months treatment 2 months follow-up Comparison of baseline, post-treatment and follow-up Headache diary recording	All received a maximum of 2 treatments per week Cervical manipulation by chiropractor (*n* = 30) (11M, 19F) Cervical manipulation by physician or physiotherapist (*n* = 27) (14M, 13F) Cervical mobilization by physiotherapist or physician (*n* = 28) (8M, 20F) Drop outs (*n* = 3)	No statistically significant difference were found between the three groups The mean reduction in attack frequency, intensity and duration pre- and post treatment were 40, 43 and 36% in the first cervical manipulation group, 13, 12 and 8% in the second cervical manipulation group and 34, 15 and 20% in the cervical mobilization group. No statistically significant effect differences were found between the three groups
Australia [12]	1980	See above 9.7 mean migraine attacks within 2 months	84 volunteers	Follow-up at 20 months post trial (see above) by a questionnaire	All received a questionnaire Drop outs (*n* = 11)	The mean reduction in attack frequency from pre trial to 20 months post trial follow-up was 58, 29 and 54% in the cervical manipulation by chiropractor, cervical manipulation group by physiotherapist or physician and the cervical mobilization group by physiotherapist or physician
USA [13]	1998	Migraineurs with at least 4 headache days per month for at least 1 year diagnosed by chiropractor	218 volunteers (46M, 172F) Age 18–65 years Mean age 38 years	A RCT of 4 months duration, i.e., 1 month baseline 2 months treatment 1 month follow-up Comparison of baseline, post-treatment and follow-up Headache diary recording	CSMT (*n* = 77) by diversified technique. A total of 14 treatments over a 8 weeks period Amitriptyline (*n* = 70). Initial dose 25 mg/day was increased weekly by 25 up to 100 mg/day. Patients were seen three times during the 2 months period. Combined CSMT and Amitriptyline (*n* = 71) Drop outs (n = 59)	Mean intensity was reduced from baseline to last 4 weeks treatment and from baseline to 4 weeks post-treatment by 40 and 42% in the CSMT group, 49 and 24% in the amitriptyline group and 41 and 25% in the combined CSMT and amitriptyline group Mean frequency was reduced from baseline to last 4 weeks treatment and from baseline to 4 weeks post-treatment by 32 and 33% in the CSMT group, 48 and 22% in the amitriptyline group and 39 and 22% in the combined CSMT and amitriptyline group
Australia [14]	2000	Migraineurs diagnosed by a questionnaire followed by diagnoses by chiropractor At least one migraine attack per month Mean migraine attack were 7.2 per months	127 volunteers (39M, 86F, 2?) Age 10–70 years Mean age 39 years	A RCT of 6 months duration, i.e., 2 months baseline 2 months treatment 2 months follow-up Comparison of baseline, post-treatment and follow-up Headache diary recording	CSMT (*n* = 83) (25M, 59F) 2 months of diversified technique, maximum of 16 sessions Control group (*n* = 40) (14M, 27F) Detuned interferential therapy Drop outs (*n* = 4)	The average response was statistically significantly better in the CSMT than the control group regarding migraine frequency (*p* < 0.005), duration (*p* < 0.01), disability (*p* < 0.05), and reduction in medication use (*p* < 0.001) The frequency and duration was reduced from baseline to follow-up by 35 and 40% in the CSMT group, and 17 and 20% in the control group

### Massage therapy

An American study included 26 participants with chronic migraine diagnosed by questionnaire [8]. Massage therapy had a statistically significant effect on pain intensity as compared with controls. Pain intensity was reduced 71% in the massage group and unchanged in the control group. Interpretation of the data is otherwise difficult and results on migraine frequency and duration are missing.

A New Zealand study included 48 migraineurs diagnosed by questionnaire [9]. The mean duration of a migraine attack was 47 h, and 51% of the participants had more than one attack per month. The study included a 3 week follow-up period. The migraine frequency was significantly reduced in the massage group as compared with the control group, while the intensity of attacks was unchanged. Results on migraine duration are missing. Medication use was unchanged, while sleep quality was significantly improved in the massage group (*p* < 0.01), but not in the control group.

### Physical therapy

An American physical therapy study included female migraineurs with frequent attacks diagnosed by a neurologist according to the criteria of the International Headache Society [3, 10]. Clinical effect was defined as >50% improvement in headache severity. Clinical effect was observed in 13% of the physical therapy group and 51% of the relaxation group (*p* < 0.001). The mean reduction in headache severity was 16 and 41% from baseline to post-treatment in the physical therapy and relaxation groups. The effect was maintained at 1 year follow-up in both groups. A second part of the study offered persons without clinical effect in the first part of the study, the other treatment option. Interestingly, clinical effect was observed in 55% of those whom received physical therapy in the second round who had no clinical effect from relaxation, while 47% had clinical effect from relaxation in the second round. The mean reduction in headache severity was 30 and 38% in the physical therapy and relaxation groups. Unfortunately, the study did not include a control group.

### Chiropractic spinal manipulative treatment

An Australian study included migraineurs with frequent attacks diagnosed by a neurologist [11]. The participants were divided into three study groups; cervical manipulation by chiropractor, cervical manipulation by physiotherapist or physician, and cervical mobilization by physiotherapist or physician. The mean migraine attack duration was skewed in the three groups, as it was much longer in cervical manipulation by chiropractor (30.5 h) than cervical manipulations by physiotherapist or physician (12.2 h) and cervical mobilization groups (14.9 h). The study had several investigators and the treatment within each group was beside the mandatory requirements free for the therapists. No statistically significant differences were found between the three groups. Improvement was observed in all three groups post-treatment (Table 3). Prior to the trial, chiropractors were confident and enthusiastic about the efficacy of cervical manipulation, while physiotherapists and physicians were doubtful about the relevance. The study did not include a control group although cervical mobilization is mentioned as the control group in the paper. A follow-up 20 months after the trial showed further improvement in the all three groups (Table 3) [12].

An American study included 218 migraineurs diagnosed according to the criteria of the International Headache Society by chiropractors [13]. The study had three treatment groups, but no control group. The headache intensity on days with headaches was unchanged in all three groups. The mean frequency was reduced equally in the three groups (Table 3). Over the counter (OTC) medication was reduced from baseline to 4 weeks post-treatment with 55% in the CSMT group, 28% in the amitriptyline group and 15% in the combined CSMT and amitriptyline group.

The second Australian study was based on questionnaire diagnoses on migraine [14]. The participants had migraine for mean 18.1 years. The effect of CSMT was significant better than the control group (Table 3). The mean reduction of migraine frequency, intensity and duration from baseline to follow-up were 42, 13, and 36% in CSMT group, and 17, 5, and 21% in the control group (data calculated by the reviewers based on figures from the paper).

## Discussion

### Methodological considerations

The prevalence of migraine was similar based on a questionnaire and a direct physician conducted interview, but it was due to equal positive and negative misclassification by the questionnaire [15]. A precise headache diagnosis requires an interview by a physicians or other health professional experienced in headache diagnostics. Three of the seven RCTs ascertained participants by a questionnaire, with the diagnostic uncertainty introduced by this (Table 3).

The second American study included participants with at least four headache days per months [13]. The mean headache severity on days with headache at baseline varied from 4.4 to 5.0 on a 0–10 box scale in the three treatment groups. This implies that the participants had co-occurrence of tension-type headache, since tension-type headache intensity usually vary between 1 and 6 (mild or moderate), while migraine intensity can vary between 4 and 9 (moderate or severe), but usually it is a severe pain between 7 and 9 [16, 17]. The headache severity on days with headache was unchanged between baseline and at follow-up, indicating that the effect observed was not exclusively due to an effect on migraine, but also an effect on tension-type headache.

RCTs that include a control group are advantageous to RCTs that compare two active treatments, since the effect in the placebo group rarely is zero and often varies. An example is RCTs on acute treatment of migraine comparing the efficacy of subcutaneous sumatriptan and placebo showed placebo responses between 10 and 37%, while the therapeutic effect, i.e., the efficacy of sumatriptan minus the efficacy of placebo was similar [18, 19]. Another example is a RCT on prophylactic treatment of migraine, comparing topiramate and placebo [20]. The attack reduction increased along with increasing dose of topiramate 50, 100 and 200 mg/day. The mean migraine attack frequency was reduced from 1.4 to 2.5 attacks per month in the topiramate groups and 1.1 attacks per month in the placebo group from baseline, with mean attack frequencies varying from 5.1 to 5.8 attacks per month in the four groups.

Thus, interpretation of the efficacy in the four RCTs without a control group is not straight forward [9–12]. The methodological quality of all seven RCTs had room for improvement as the maximum score 100 was far from expectation, especially a precise migraine diagnosis is important.

Several of the studies relatively include a few participants, which might cause type 2 errors. Thus, power calculation prior to the study is important in the future studies. Furthermore, the clinical guidelines from the International Headache Society should be followed, i.e., frequency is a primary end point, while duration and intensity can be secondary end points [21, 22].

### Results

The two RCTs on massage therapy included relatively a few participants, along with shortcomings mentioned in Table 3 [8, 9]. Both studies showed that massage therapy was significantly better than the control group, by reducing migraine intensity and frequency, respectively. The 27–28% (34–7% and 30–2%) therapeutic gain in migraine frequency reduction by massage therapy is comparable with the 6, 16 and 29% therapeutic gain in migraine frequency reduction by prophylactic treatment with topiramate 50, 100 and 200 mg/day [20].

The single study on physiotherapy is large, but do not include a control group [10]. The study defined responders to have 50% or more reduction in migraine intensity. The responder rate to physical therapy was only 13% in the first part of the study, while it was 55% in the group that did not benefit from relaxation, while the responder rate to relaxation was 51% in the first part of the study and 47% in the group that did not benefit from physical therapy. A reduction in migraine intensity often correlates with reduced migraine frequency. For comparison, the responder rate was 39, 49, 47 and 23% among those who received topiramate 50, 100 and 200 mg/day and placebo as defined by 50% or more reduction in migraine frequency [20]. A meta-analysis of 53 studies on prophylactic treatment with propranolol showed a mean 44% reduction in migraine activity [23]. Thus, it seems that physical therapy and relaxation has equally good effect as topiramate and propranolol.

Only one of the four RCTs on chiropractic spinal manipulative therapy (CSMT) included a control group, while the other studies compared with other active treatment [11–14]. The first Australian study showed that the migraine frequency was reduced in all three groups when baseline was compared with 20 months post trail [11, 12]. The chiropractors were highly motivated to CSMT treatment, while physicians and physiotherapist were more sceptical, which might have influenced on the result. An American study showed that CSMT, amitriptyline and CSMT + amitriptyline reduced the migraine frequency 33, 22 and 22% from baseline to post-treatment (Table 3). The second Australian study found that migraine frequency was reduced 35% in the CSMT group, while it was reduced 17% in the control group. Thus, the therapeutic gain is equivalent to that of topiramate 100 mg/day and the efficacy is equivalent to that of propranolol [20, 23].

Three case reports raise concerns about chiropractic cervical SMT, but a recent systematic review found no robust data concerning the incidence or the prevalence of adverse reactions following chiropractic cervical SMT [24–27]. When to refer migraine patients to manual therapies? Patients not responding or tolerating prophylactic medication or who wish to avoid medication for other reasons, can be referred to massage therapy, physical therapy or chiropractic spinal manipulative therapy, as these treatments are safe with a few adverse reactions [27–29].

## Conclusion

Current RCTs suggest that massage therapy, physiotherapy, relaxation and chiropractic spinal manipulative therapy might be equally efficient as propranolol and topiramate in the prophylactic management of migraine. However, a firm conclusion requires, in future, well-conducted RCTs without the many methodological shortcomings of the evaluated RCTs on manual therapies. Such studies should follow clinical trial guidelines from the International Headache Society [21, 22].
